# A Transcriptional Signature of IL-2 Expanded Natural Killer Cells Predicts More Favorable Prognosis in Bladder Cancer

**DOI:** 10.3389/fimmu.2021.724107

**Published:** 2021-11-10

**Authors:** Yuhan Sun, Alexander James Sedgwick, Md Abdullah-Al-Kamran Khan, Yaseelan Palarasah, Stefano Mangiola, Alexander David Barrow

**Affiliations:** ^1^Department of Microbiology and Immunology, The University of Melbourne and The Peter Doherty Institute for Infection and Immunity, Melbourne, VIC, Australia; ^2^Institute of Molecular Medicine, University of Southern Denmark, Odense, Denmark; ^3^Division of Bioinformatics, Walter and Eliza Hall Institute, Parkville, VIC, Australia

**Keywords:** NK cell, bladder cancer, prognosis, NK receptors, TCGA, anti-tumor immunity

## Abstract

Activation of natural killer (NK) cell function is regulated by cytokines, such as IL-2, and secreted factors upregulated in the tumor microenvironment, such as platelet-derived growth factor D (PDGF-DD). In order to elucidate a clinical role for these important regulators of NK cell function in antitumor immunity, we generated transcriptional signatures representing resting, IL-2-expanded, and PDGF-DD-activated, NK cell phenotypes and established their abundance in The Cancer Genome Atlas bladder cancer (BLCA) dataset using CIBERSORT. The IL-2-expanded NK cell phenotype was the most abundant in low and high grades of BLCA tumors and was associated with improved prognosis. In contrast, *PDGFD* expression was associated with numerous cancer hallmark pathways in BLCA tumors compared with normal bladder tissue, and a high tumor abundance of *PDGFD* transcripts and the PDGF-DD-activated NK cell phenotype were associated with a poor BLCA prognosis. Finally, high tumor expression of transcripts encoding the activating NK cell receptors, KLRK1 and the CD160–TNFRSF14 receptor–ligand pair, was strongly correlated with the IL-2-expanded NK cell phenotype and improved BLCA prognosis. The transcriptional parameters we describe may be optimized to improve BLCA patient prognosis and risk stratification in the clinic and potentially provide gene targets of therapeutic significance for enhancing NK cell antitumor immunity in BLCA.

## Introduction

Bladder cancer (BLCA) is a disease of the elderly in the developed world (1, 2). An aging population, industrialization, and endemic tobacco smoking in developing nations mean that global BLCA diagnoses are estimated to double (2). The first-line management of high-grade non-muscle-invasive BLCA involves transurethral resection of the bladder tumor (3) and administration of an induction course of intravesical bacille Calmette–Guerin (BCG) vaccine. Longer-term BCG maintenance therapy retards the progression and recurrence of BLCA disease (4, 5), but toxicity and intolerance, albeit rare, confer considerable risks to BLCA patients (6, 7).

Transition to a BCG-refractory high-grade BLCA is associated with poor survival outcomes (8), and radical cystectomy (RC) for patients that fail intravesical immunotherapy is the current gold standard treatment (9). Nonetheless, RC and chemotherapy are costly (10), highly invasive, and are associated with significant side effects (11) and impaired quality of life (12). Limited therapeutic options beyond systemic chemotherapy have resulted in dire outcomes for patients with metastatic BLCA disease (11, 13). Thus, there is an urgent need for less invasive, tolerable, and durable alternatives for intractable BLCA.

Early incursions into immune checkpoint blockade (ICB) in BLCA have yielded promising results (14–19). However, only 30% of bladder cancer patients with metastatic disease respond to ICB therapy. To circumvent tumor incompatibility (20) and acquired resistance to ICB (21), a more detailed characterization of tumor immune surveillance pathways will undoubtedly inform more effective immunotherapies for BLCA patients (22–25). Natural killer (NK) cells are innate lymphocytes that produce IFN-γ but are distinct from other innate lymphoid cells because they specialize in the cytolysis of malignant and infected cells and are thus considered the innate counterparts of cytotoxic T lymphocytes (26). While NK cell cytotoxicity is known to contribute to BCG therapeutic benefit (27, 28), immune surveillance of malignant uroepithelial tissue by NK cells is understudied (29).

NK cell cytotoxicity is regulated by antagonistic signaling networks moderated by an array of activating and inhibitory cell surface receptors (30, 31). The downregulation of ligands, such as MHC-I, for inhibitory receptors in conjunction with the abundant expression of ligands recognized by activating receptors, such as killer cell lectin-like receptor K1 (KLRK1) (32) and the natural cytotoxicity receptors (NCRs) (33), predisposes tumor cells to NK cell elimination (34). Human KLRK1, also known as NKG2D, is expressed by NK cells and CD8^+^ T lymphocytes and recognizes a range of stress-inducible ligands expressed on malignant cells, such as MICA, MICB, and the ULBP-binding proteins 1–6 that are collectively known as “NKG2D ligands” (NKG2D-L) (35, 36). Indeed, *in vitro* assays suggest that NKG2D recognition of stress-inducible ligands is a prominent mode of BLCA tumor cell recognition (37). In contrast, the NCR NKp44 can bind to platelet-derived growth factor D (PDGF-DD), which is overexpressed by many solid tumors including BLCA, and may activate NK cell antitumor functions to control tumor growth (38). NK cells express many other activating and inhibitory receptors that are likely to cooperate to elicit maximal NK cell activity in the tumor microenvironment (39). In-depth analysis of the immune cell phenotypes and receptors associated with improved BLCA patient prognosis will shed light on the tumor surveillance pathways that may be enhanced for improved BLCA immunotherapy (40).

Like NKG2D, TNFRSF14 is expressed by NK cells and CD8^+^ T lymphocytes and has multiple ligands, such as TNFSF14 (also known as LIGHT), lymphotoxin-α (LTA), CD160 (also known as natural killer cell receptor BY55), and B- and T-lymphocyte-associated protein (BTLA). TNFRSF14, also known as herpes virus mediator of entry (HVEM), can convey either lymphocyte activation or inhibition depending on *cis* and *trans* interactions with the ligand (41). For example, acting as a receptor for TNFSF14 or LTA, TNFRSF14 can stimulate downstream NF-κB signaling to promote NK cell and T-lymphocyte proliferation, IFN-γ production, and tumor cell clearance (42–45). CD160 is also expressed by NK cells and CD8^+^ T cells as GPI-anchored and transmembrane forms. Binding of TNFRSF14 to the transmembrane form of CD160 delivers an activating signal that can promote NK cell cytotoxicity and IFN-γ production (46). In contrast, TNFRSF14 binding *in cis* to BTLA inhibits *trans* interactions with LIGHT, LTA, or CD160, to maintain NK cells and T lymphocytes in a resting state, thus tuning lymphocyte activation to the surrounding tumor microenvironment (47). Interestingly, TNFRSF14 is also expressed by tumor cells and TNFRSF14 ligation can inhibit bladder cancer cell proliferation by inducing apoptosis (48).

Here, we investigated the clinical impact of the abundance of resting, IL-2-expanded, and PDGF-DD-activated NK cell phenotypes and the receptors they express in the BLCA tumor microenvironment. To achieve this, we generated transcriptional signatures representing the latter NK cell activation states to estimate their relative abundance in The Cancer Genome Atlas (TCGA) BLCA dataset and tested the association with curated progression-free survival (49).

## Methods

### Material Availability

The R codes for the analyses presented in this study are available at RAGG3D/BLCA_IL2NK (github.com).

### Data Collection and Validation of Functional NK Cell Datasets

Gene transcript-abundance and patient clinical information were collected from TCGA through the GDC Data Portal (50). Progression-free survival information was used as a measure of clinical outcome (49). The cell-type-specific transcriptional signatures were derived from a large collection of RNA sequencing samples spanning a wide range of cell types. For NK cells, an experimentally derived dataset for IL-2-expanded [27 biological replicates (38)], PDGF-DD activated *via* NKp44 signaling [4 biological replicates (38)] and resting (25 biological replicates from six datasets) were included. For resting NK cells and other cell types, the data collected were from the following datasets: BLUEPRINT (51), Monaco et al. (52), ENCODE (53), Squires et al. (54), GSE77808 (55), Tong et al. (56), PRJNA339309 (57), GSE122325 (58), FANTOM5 (59), GSE125887 (60), GSE130379 (61), and GSE130286 (62).

In order to validate the functional status of the RNA-seq datasets curated in this study, we determined the expression of transcripts for surface proteins commonly upregulated during IL-2 expansion of NK cells, such as CD69, CD25, CD70, and NKp44 (63–67). With the exception of *CD69*, transcripts for *IL2RA*, *CD70*, and *NCR2* were significantly upregulated in IL-2-expanded and PDGF-DD-activated NK cells compared with resting NK cells (**Supplementary Figure 1**), which we conclude sufficiently validates the use of these curated datasets to generate transcriptional signatures representative of “resting” and “IL-2-expanded” NK cell phenotypes.

### Generation of Transcriptional Signatures

In order to derive transcriptional signatures of 24 cell types (memory B cell, naive B cell, immature dendritic myeloid cell, mature dendritic myeloid cell, endothelial, eosinophil, epithelial, fibroblast, macrophage M1 and M2, mast cell, monocyte, neutrophil, resting NK cells, IL-2-expanded NK cells, PDGF-DD-activated NK cells, central memory CD4 T cell, effector memory CD4 T cell, central memory CD8 T cell, effector memory CD8 T cell, naive CD8 T cell, gamma-delta T cell, helper T cell, and regulatory T cell), a total of 592 highly curated (i.e., for which identity was confirmed in the literature), non-redundant biological replicates (including 25 resting NK cell samples, 27 IL-2-expanded NK cell samples, and 4 PDGF-DD-activated NK cell samples) have been used. Due to the sparse nature of a heterogeneous set of datasets, the expected value and variability of gene transcription abundance was inferred for each cell type using a publicly available Bayesian statistical model (github: stemangiola/cellsig), based on a negative binomial data distribution (68). This model allows to fit sparse data (e.g., transcript abundance of one gene for which data are available in a subset of reference biological replicates) and calculate theoretical data distributions of cell-type/gene pairs. The cell-type transcriptional marker selection was based on the pairwise comparison of each cell type within cell-type categories along a cell differentiation hierarchy (**Supplementary Figure 2**) (69). For example, all cell-type permutations from the root node of level 1 (including epithelial, endothelial, fibroblasts, and immune cells) were interrogated in order to select the genes for which the transcript abundance distribution (data generated from the posterior distribution) was higher for one cell type compared with another. This was executed calculating the distance of the upper and lower 95% credible intervals, respectively (obtained from cellsig). From each comparison, the top 5, 10, and 20 genes per cell-type pair were selected from levels 1, 2, and 3, respectively (**Supplementary Figure 2**). The marker gene list is composed by the union of genes for all levels. This hierarchical approach favors the identification of marker genes that distinguish broad cell-type categories as well as specific activation phenotypes.

The top marker genes (upregulated) that segregate IL-2-expanded NK cells from PDGF-DD-activated NK cells are ERP29, IMPDH2, and MFSD10. The top markers (upregulated) that segregate activated from resting NK cells are RPSAP9, POTEF, POTEE, GOLGA8IP, HERC2P4, and HNRNPA3P1. The top markers (upregulated) that segregate NK cells from other major immune cells are CD247, CTSW, HOPX, GZMA, ID2, IL2RB, SHROOM1, CD74, NKG7, CATSPER1, CCNJL, MTRNR2L6, CST7, EIF4A1, KRT81, PPP1R9A, and SH2D2A. The full signature matrix is provided as supplementary material (**Supplementary File 1**).

### Benchmark of the Transcriptional Signatures

To test whether the selected signature for IL-2-expanded NK cells was suitable to accurately detect changes in cell abundance in association with progression-free survival, we implemented a benchmark on simulated tissue mixtures. This benchmark was organized in 81 simulation conditions, each of which included 63 test runs. The simulation conditions were i) the amount of tissue mixture (replicates) from 250 to 1,000, ii) the degree of change (slope) from −1 to 1, and iii) the proportion of a foreign cell type, whose signature was not included in our reference (i.e., neural cells), from 0 to 0.8 (80%). For each test run, a number of tissue mixtures (replicates) were simulated. One mixture is created as the sum of the transcriptional profiles for each cell type, weighted by a proportion array (summing to one) that represents the relative amount of cell types within the tissue. The transcriptional profiles were samples at random from our reference database. The proportion arrays (for each run) were built according to a linear model, correlating the cell-type proportion with progression-free survival. For example, in case T cells were to be positively associated with progression-free survival, the tissue mixtures (i.e., patients) with bigger progression-free survival would be characterized by a larger proportion of T cells. The values of progression-free survival were simulated according to real-world data. We sampled the progression-free survival time from the TCGA BLCA patient cohort. For each test run (including several simulated mixtures), only one cell type was set up as being associated with progression-free survival.

The cell-type proportion associations were estimated for each test run. The estimation included two steps: deconvolution and Cox regression of the estimated cell-type proportions. To simulate censored data (partial follow-up for progression-free survival time), the Cox regression was provided with halved the time-to-event for half of the tissue mixtures. We classified IL-2-expanded NK cells as changing or not-changing based on a *p*-value threshold of 0.05. The framework tidybulk was used to infer the cell-type proportions through CIBERSORT and perform a multiple Cox regression on the predicted proportions (logit-transformed) (70), with progression-free survival censored time as a covariate. The significance calls were compared with the ground truth to generate a receiver operating characteristic (ROC) curve.

### Estimation of the Association of Cell-Type Abundance With Relapse-Free Patient Survival

To estimate the cell-type abundance for each biological replicate, we used CIBERSORT with our RNA sequencing-derived gene marker signature. In order to estimate the clinical relevance of NK activation phenotypes, we produced a Kaplan–Meier estimator (71) based on the median proportion split of each cell type. Percent survival *vs.* time-to-event statistics were calculated by the log-rank (Mantel–Cox) test (72). Statistics of Kaplan–Meier curves were performed by the log-rank test then adjusted by the Benjamini–Hochberg (BH) procedure. A table of all *p*-values prior to adjustment is provided in **Supplementary Table 1**. Data analysis was performed using the R environment in R Studio (73). Packages include tidyverse (74), tidybulk (75), survminer (76), survival (70, 77), foreach (78), org.Hs.eg.db (79), cowplot (80), ggsci (81), GGally (82), gridExtra (83), grid (73), reshape (84), Hmisc (85), tidyHeatmap (86), and viridis (87).

### Functional Enrichment Analysis

To identify the unique protumorigenic pathways associated with *PDGFD* expression in BLCA tumors compared with normal bladder tissue, we utilized the functional enrichment analysis for the top 1,000 co-expressed genes of *PDGFD* in both TCGA BLCA tumors and TCGA BLCA normal tissues datasets, which we obtained from the GEPIA2 web server (88). The comparative functional enrichment analysis was performed in Gitools v1.8.4 (89) utilizing the modules constructed from the Gene Ontology (90) Biological Process (GOBP), Bioplanet pathways (91), KEGG pathways (92), Reactome pathways (93), and Wikipathways (94) databases. During the analysis, the resultant *p*-values of the enriched terms were adjusted using the multiple test correction approach outlined in the Benjamini–Hochberg’s false discovery rate (FDR) method, and we only considered those enriched pathways/terms significant which have an FDR *q*-value <0.05. From the enrichment results, we sorted and grouped the significant pathways/terms manually based on the associated protumorigenic hallmarks and immune responses.

## Results

### IL-2-Expanded NK cells Are Associated With a More Favorable BLCA Prognosis

**Figure 1:**
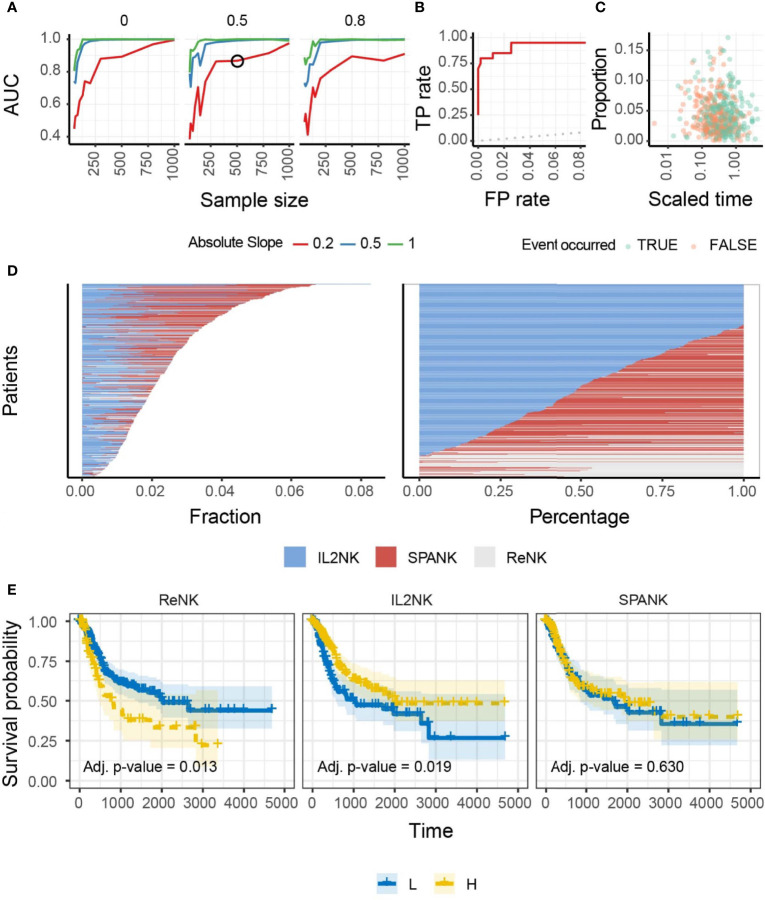
Benchmark, overall abundance and survival associations of NK phenotypes in BLCA. **(A)** Accuracy of the inference of changes in proportion of IL-2-expanded NK cells from simulated mixtures. The detection of a significant proportional change when it exists in the simulation defined a true-positive. Data points represent the area under the curve (AUC) for mixtures created with a specific combination of sample size (*x*-axis), degree of change (slope; color-coded), and proportion of foreign cell type (facets; i.e., neurons, for which we do not include transcriptional profile in the training data). A simulation condition that represents the associations we detected in the TCGA database is circled. **(B)** Receiver operating characteristic (ROC) curve, measuring the accuracy (true-positive and false-positive) for the simulated mixture circled in **(A)**. A curve touching the top-left corner (0 false-positive and 1 true-positive rates) would represent the best achievable performance. A curve overlapping the dotted line (45°) would represent a random detection of proportional changes. **(C)** The underlying association between cell type and time-to-event (e.g., survival days) of the simulated dataset circled in **(A)**. Data points represent cell-type/sample pairs. **(D)** Abundance of NK cell phenotypes (fraction and percentage) for TCGA BLCA cohort; IL2NK is the most abundant NK cell phenotype in BLCA. **(E)** Kaplan–Meier survival curve for all three NK cell phenotypes for TCGA-BLCA; high tumor abundance of IL2NK, but not the ReNK or SPANK phenotypes, is associated with a favorable BLCA patient outcome (*x*-axis, days; *y*-axis, progression-free survival).

We hypothesized that NK cells of unique phenotype can infiltrate different cancer types to confer antitumor immunity. To answer this question, we performed a benchmark for the inference of changes in the abundance of IL-2-expanded NK cells in artificial tissue mixtures built from our reference dataset (see *Methods*) to determine the ability of the IL-2-expanded NK cell signature to provide an identifiable biologically relevant signal from whole tissue RNA sequencing data (**Figure 1A**). This benchmark showed a high accuracy (area under the curve) across simulation settings including magnitude of variability, sample size, and proportion of unknown cells (please see *Methods*). An accuracy of 0.75 was reached for simulation settings that match our findings on TCGA data (slope and sample size; **Figures 1B, C****)**. These data show that our NK cell signatures have the potential to uncover clinically relevant associations from TCGA-derived whole tissue RNA sequencing data. We then defined marker genes for transcriptional signatures representing resting NK cells (ReNK) (95), IL-2-expanded NK cells (IL2NK), and a signature of PDGF-DD-activated NK cells (SPANK) (96), respectively, and established the transcript abundance of these NK cell phenotypes in TCGA BLCA cohort using CIBERSORT (please see *Methods*). Using this approach, we found that the IL2NK phenotype was more abundantly expressed in BLCA tumors compared with the ReNK or SPANK phenotypes (**Figure 1D**). Interestingly, the IL2NK phenotype, but not the ReNK or SPANK, was associated with improved BLCA patient prognosis (**Figure 1E**). In contrast to IL2NK, high tumor abundance of the ReNK phenotype was associated with poor prognosis, while abundance of the SPANK was not associated with prognosis (**Figure 1E**). The tumor abundance of signature T-cell phenotypes was also not associated with prognosis (**Supplementary Figure 3**). These results show that a high infiltration of the IL2NK phenotype in BLCA tumors is associated with improved BLCA prognosis.

### Abundance of NK Cell Phenotypes in Different Clinical Grades of BLCA Tumors

**Figure 2:**
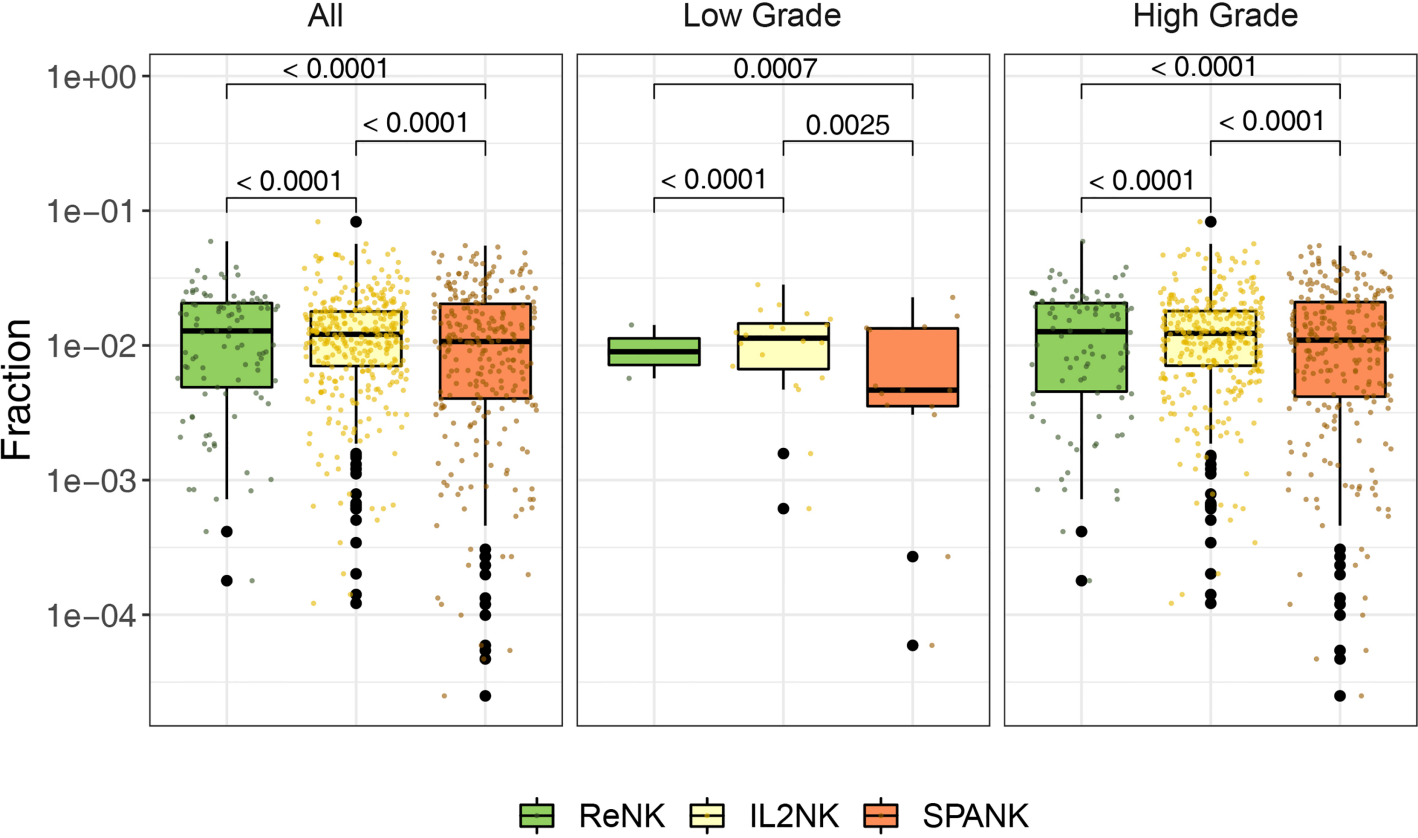
Abundance of NK cell phenotypes in different clinical BLCA grades. Abundance of the ReNK, IL2NK, and SPANK NK cell phenotypes (log10 transformed fraction) for TCGA BLCA cohort partitioned into low and high clinical grades. IL2NK is the most abundant NK cell phenotype in low- and high-grade BLCA tumors, followed by the SPANK and then ReNK phenotypes. Wilcoxon signed-rank test was conducted to examine the differences between clinical grades. P-values were adjusted by Benjamini-Hochberg procedure.

Since a high tumor abundance of the IL2NK phenotype was associated with a more favorable BLCA prognosis, we next asked whether a particular NK cell phenotype was preferentially associated with a different clinical grade of the BLCA tumor. BLCA tumors were partitioned into either low or high grade and the abundance of the ReNK, IL2NK, and SPANK NK cell phenotypes was determined, respectively (**Figure 2**). In both low- and high-grade BLCA tumors, the IL2NK phenotype was the most abundant followed by the SPANK and then ReNK phenotypes (**Figure 2**). Remarkably, abundance of either the ReNK, IL2NK, or SPANK NK cell phenotypes did not differ between low- and high-grade BLCA tumors (**Supplementary Figure 4**). These results show that the IL2NK phenotype is more abundant in low and high BLCA tumor grades, followed by the SPANK and then ReNK phenotypes.

### *PDGFD* Expression Is Associated With Cancer Hallmarks and Poor BLCA Prognosis

**Figure 3:**
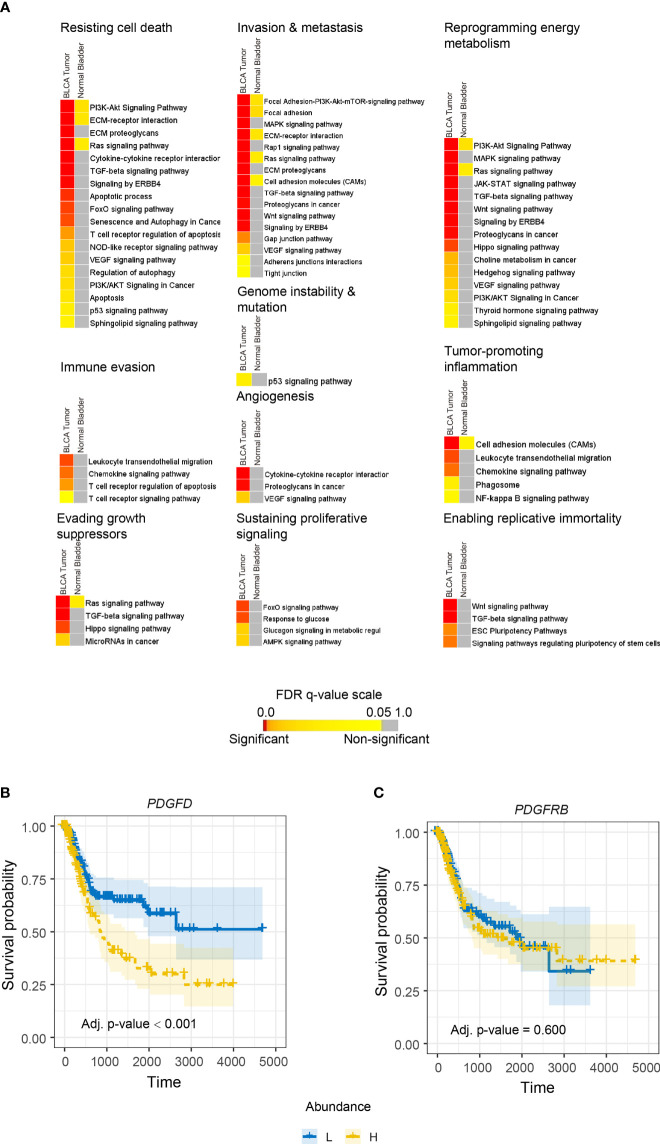
*PDGFD* expression is associated with the activation of protumorigenic pathways and poor prognosis in BLCA. **(A)** Cancer hallmark signaling pathways associated with *PDGFD* expression in BLCA tumors and normal bladder tissue. Color-coded heatmaps represent the statistical significance of the functionally enriched pathways. Color toward red indicates the most significant pathways, while the yellow color represents less significant and gray color represents the non-significant events. Only selected significant terms were presented within the heatmaps. Kaplan–Meier survival curves constructed for **(B)** *PDGFD* or **(C)** *PDGFRB* expression in BLCA tumors. High tumor expression of *PDGFD* is associated with poor BLCA prognosis (*x*-axis, days; *y*-axis, progression-free survival).

PDGF-DD is produced by many aggressive cancers and binds to PDGFR-β expressed on tumor cells to induce protumorigenic signaling pathways that are thought to be associated with poor patient outcome (38–42, 97). Even though the SPANK and IL2NK represent activated NK cell phenotypes, our initial analysis showed that tumor abundance of the SPANK was not associated with BLCA patient prognosis, unlike IL2NK (**Figure 1B**). We hypothesized that protumorigenic pathways associated with *PDGFD* expression might mask any antitumor functions of PDGF-DD-activated NK cells on BLCA patient prognosis. We downloaded the top 1,000 transcripts from the GEPIA2 website associated with *PDGFD* expression in BLCA and in normal bladder tissue (please see *Methods*) and performed gene enrichment analysis to identify enriched protumorigenic pathways (**Figure 3**). Many protumorigenic pathways associated with *PDGFD* expression that represent core cancer hallmarks (98, 99) were enriched in BLCA but not in normal bladder tissue (**Figure 3A**). These pathways include PDGF signaling and response to growth factor stimulus (**Supplementary Figures 5A, B****)**. These data show that protumorigenic pathways representing key cancer hallmarks are associated with *PDGFD* expression in BLCA tumors.

Since protumorigenic pathways associated with *PDGFD* expression were enriched in BLCA, we next determined the relationship between tumor expression of *PDGFD* or *PDGFRB* and BLCA patient prognosis (**Figure 3**). High tumor expression of *PDGFD* was associated with a poor BLCA prognosis compared with those BLCA patients with low tumor expression of *PDGFD* (**Figure 3A**). In contrast, tumor expression of *PDGFRB* did not influence BLCA patient prognosis (**Figure 3B**). Overall, these data show that a high tumor expression of *PDGFD* is associated with the activation of core cancer hallmarks and poor BLCA patient prognosis.

### Stratifying Tumors Based on *PDGFD* Expression Reveals the SPANK Phenotype Is Associated With a Poor BLCA Prognosis

**Figure 4:**
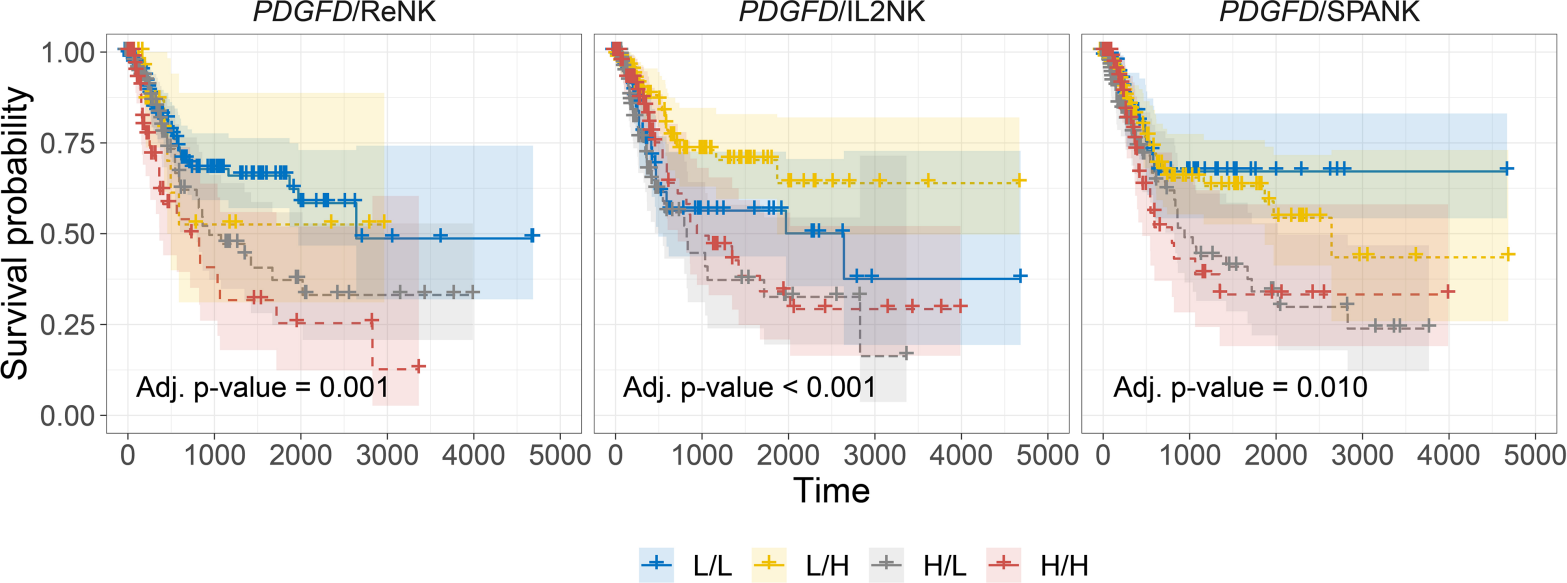
Tumor abundance of IL2NK, but not the SPANK, counteracts the protumorigenic effects of *PDGFD* on BLCA patient prognosis. Combined BLCA patient survival analysis stratified for tumor expression (median split) of *PDGFD* and each NK cell phenotype, ReNK, IL2NK, and SPANK, respectively. KM curves display the survival of BLCA patients plotted in all four combinations for each stratum, respectively (L/L, L/H, H/L, and H/H). Low *PDGFD* expression and high IL2NK abundance in BLCA tumors are associated with improved prognosis, whereas low expression of *PDGFD* and abundance of the SPANK phenotype in BLCA tumors are associated with a poor prognosis (*x*-axis, days; *y*-axis, progression-free survival).

In addition to the activation of protumorigenic pathways, PDGF-DD can evoke NK cell antitumor functions through binding to NKp44 and signaling *via* the associated DAP12 adaptor (38, 64, 100). In support of this, the DAP12 signaling pathway was strongly associated with *PDGFD* expression in BLCA tumors (**Supplementary Figures 5C, D****)**. However, the contribution of each NK cell phenotype in mitigating the detrimental effect of *PDGFD* expression on patient prognosis remained unclear (**Figure 3A**). We next determined the association between each NK cell phenotype and patient prognosis for BLCA tumors stratified for *PDGFD* expression. When BLCA tumors were stratified for high *PDGFD* expression, high tumor abundance of the ReNK was associated with poor prognosis, and neither the IL2NK nor SPANK phenotypes were associated with patient prognosis, underscoring the strong association between high BLCA tumor expression of *PDGFD* and poor patient prognosis (**Figure 4**). In contrast, when BLCA tumors were stratified for low *PDGFD* expression, a high tumor abundance of IL2NK was associated with improved prognosis, whereas the SPANK was associated with poor prognosis and to a lesser extent the ReNK (**Figure 4**). These results show that a high tumor abundance of IL2NK in BLCA tumors with low *PDGFD* expression is associated with a more favorable prognosis, whereas a high tumor abundance of the ReNK or SPANK phenotypes is associated with poor BLCA prognosis.

### Critical Role for IL2NK-Associated NK Cell Receptors in BLCA Prognosis

**Figure 5:**
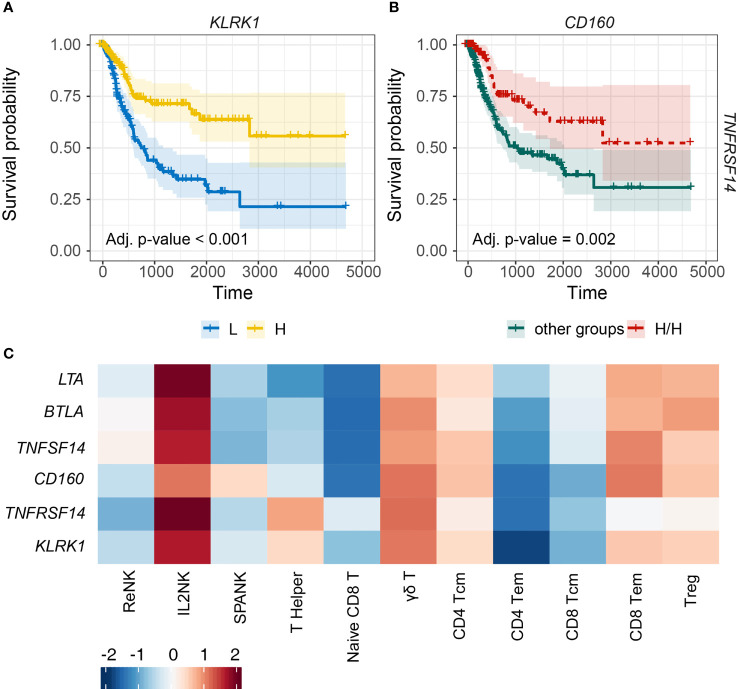
Tumor expression of activating NK cell receptors is associated with the IL2NK phenotype and a more favorable BLCA prognosis. **(A)** KM plots displaying progression-free survival of BLCA patients stratified for tumor expression (median split) of *KLRK1* (*x*-axis, days; *y*-axis, progression-free survival). **(B)** Combined BLCA patient survival analysis stratified for tumor expression (median split) for *CD160* and *TNFRSF14*. KM curves display the survival of BLCA patients plotted for the highest strata of expression for *CD160* and *TNFRSF14* (H/H) compared with all other strata combinations for *CD160* and *TNFRSF14* combined. High tumor abundance of *CD160* and *TNFRSF14* is associated with improved BLCA prognosis (*x*-axis, days; *y*-axis, progression-free survival). **(C)** Heatmap displaying correlations between the expression of each NK cell receptor transcript (*y*-axis) and immune cell phenotype (x-axis), respectively (Tcm, central memory T cell; Tem, effector memory T cell; Treg, regulatory T cell).

NK cells express an array of activating and inhibitory cell surface receptors, but how these NK receptors function for effective antitumor immunity in different types of cancer remains unclear. Since the IL2NK phenotype was associated with improved prognosis, we next determined whether tumor expression of specific NK cell receptors was critical for BLCA patient prognosis (**Figure 5**). BLCA tumors with high expression of *KLRK1*, which encodes the activating NKG2D receptor, had a much improved prognosis compared with BLCA patients with low tumor expression of *KLRK1* (**Figure 5A**). Human NKG2D binds to a range of stress-inducible NKG2D-L, such as MICA, MICB, and ULBPs 1–6, and so we next determined patient prognosis for BLCA tumors stratified for *KLRK1* expression and each NKG2D-L, respectively (**Supplementary Figure 6**). When *KLRK1* expression was high in BLCA tumors, expression of each NKG2D-L did not influence patient prognosis (**Supplementary Figure 6**). However, when *KLRK1* expression was low in BLCA tumors, expression of *MICA* and *MICB* trended toward improved prognosis, whereas *ULBP1* expression trended toward poor prognosis (**Supplementary Figure 6**).

TNFRSF14, also known as herpes virus mediator of entry (HVEM), can induce lymphocyte activation or inhibition depending on *cis* or *trans* interactions with ligand. TNFRSF14 can bind to four possible ligands, TNFSF14, LTA, CD160, or BTLA. TNFRSF14 interactions with either TNFSF14, LTA, or CD160 can promote NK cell activation, whereas binding to BTLA can induce NK cell inhibition. We noted that high BLCA tumor expression of *TNFRSF14* or *CD160* trended toward improved prognosis compared with either *TNFSF14*, *LTA*, or *BTLA* (**Supplementary Figure 7**). We next determined patient prognosis for BLCA tumors stratified for the expression of *TNFRSF14* and *CD160*. High expression of both *TNFRSF14* and *CD160* in BLCA tumors was associated with more favorable patient prognosis compared with all the other groups (**Figure 5B**). Finally, expressions of the *KLRK1*, *TNFRSF14*, and *CD160* NK cell receptor genes were all positively correlated with the IL2NK phenotype compared with the ReNK or SPANK (**Figure 5C**). These results show that high tumor expression of transcripts encoding the NK cell receptors KLRK1, TNFRSF14, and CD160 may be critical for antitumor immunity in BLCA because the expression of these receptors is associated with the IL2NK cell phenotype and a more favorable BLCA prognosis.

## Discussion

The clinical relevance of NK cells in cancer immune surveillance, particularly for solid tumors, remains unclear. We hypothesized that different NK cell phenotypes may be present in diverse cancer types and the abundance of these NK cell phenotypes may be associated with prognosis. We constructed transcriptional signatures representing ReNK, IL2NK, and SPANK and used a computational approach to determine the association between the abundance of these NK cell phenotypes in BLCA tumors and patient prognosis using the TCGA cohort. Using this approach, we found that a high tumor abundance of the IL2NK phenotype was associated with improved BLCA prognosis, but not the ReNK or SPANK.

**Figure 6:**
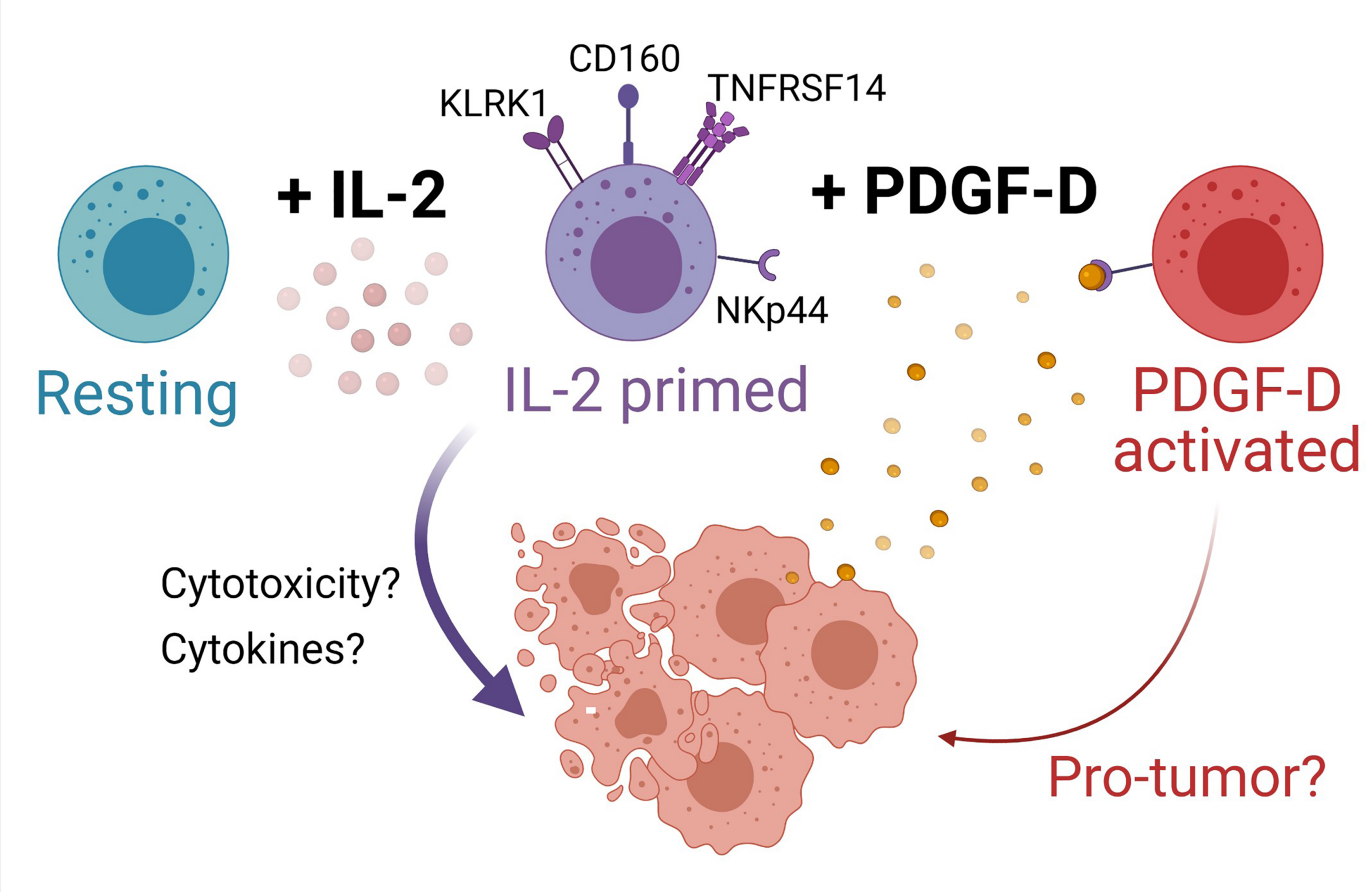
Model for NK cell surveillance of BLCA. In contrast to resting NK cells (turquoise), IL-2 (pink circles) primes NK cells (purple cells) to express the activating receptors KLRK1 (also known as NKG2D), CD160, and TNFRSF14. Engagement of NKG2D promotes NK cell cytotoxicity of BLCA tumor cells (peach cells) expressing NKG2D ligands. BLCA tumor cells can also express TNFRSF14 and binding of CD160 on activated NK cells to TNFRSF14 on tumor cells promotes apoptosis of tumor cells, thus improving BLCA patient prognosis. The binding of TNFRSF14 to CD160 on neighboring NK cells may augment NK cell activation. IL-2-primed NK cells also express NKp44, whereas resting NK cells do not. BLCA tumor cells produce platelet-derived growth factor D (PDGF-DD, yellow circles) which induces cellular activation *via* NKp44 to generate a protumorigenic NK cell phenotype (red cells) that may be detrimental for patient prognosis. Created with BioRender.com.

PDGF-DD expression is dysregulated in several cancers including BLCA and activates several protumor pathways with adverse effects on prognosis (38, 97). Analysis of the expression of transcripts encoding PDGF-D and its receptor, PDGFRβ, showed that high expression of *PDGFD* in BLCA tumors was most strongly associated with poor prognosis. Since all three NK cell phenotypes were detected in BLCA tumors, we speculated that PDGF-DD-activated NK cells might counterbalance the effect of *PDGFD* expression on BLCA prognosis. Interestingly, when BLCA tumors were stratified for *PDGFD* expression and each respective NK cell phenotype, a high tumor abundance of the SPANK phenotype was associated with a poor BLCA prognosis. Conversely, a high abundance of the IL2NK phenotype in BLCA tumors was associated with improved prognosis. Since the NKp44 receptor for PDGF-DD is upregulated by IL-2-expanded NK cells, these results suggest a critical balance between activation *via* NKp44/PDGF-DD signaling and a clinically unfavorable prognosis *versus* maintaining a high proportion of the IL-2-expanded NK cell phenotype and a clinically favorable prognosis (**Figure 6**) (38). Moreover, the ReNK phenotype was associated with a poor BLCA prognosis, and it is entirely possible that failure of NK cells to become activated, e.g., by IL-2, is detrimental for BLCA patient survival (**Figure 6**). Interestingly, other cellular and tumor ligands have been reported to bind and regulate NKp44 signaling, such as Nidogen-1 (101), Syndecan-4 (102), a subset of HLA-DP molecules (103), a splice variant of the mixed lineage leukemia 5 (MLL5) gene (104), and proliferating cell nuclear antigen (PCNA) (105). It will be interesting to determine the expression of these latter gene products in the BLCA tumor microenvironment and the influence on the associations between the NK cell phenotypes that we describe and BLCA prognosis.

We speculated that if the IL2NK phenotype was critical for NK cell surveillance of BLCA tumors and improved prognosis, then NK cell receptors would also be associated with improved BLCA prognosis. In support of this hypothesis, high tumor expression of transcripts for the *KLRK1*, *TNFRSF14*, and *CD160* NK cell receptors was associated with improved BLCA prognosis, suggesting that the expression of these NK cell receptor gene products by IL-2-expanded NK cells may be critical for BLCA antitumor immunity. High tumor expression of *KLRK1*, more commonly known as NKG2D, was strongly associated with enhanced BLCA patient prognosis. KLRK1/NKG2D is an activating receptor expressed by NK cells and CD8^+^ T cells and recognizes a range of stress-inducible ligands, such as MICA, MICB, and the ULBP-binding proteins 1–6, collectively known as “NKG2D ligands” (NKG2D-L), that are expressed on malignant and virus-infected cells. Our data and recent *in vitro* studies (37) suggest that NKG2D is a key receptor for NK cell surveillance of BLCA tumor cells.

Like NKG2D, TNFRSF14 is also expressed by NK cells and T lymphocytes and has multiple ligands; TNFSF14, LTA, and CD160 activate TNFRSF14 signaling and NK cell antitumor functions, whereas BTLA inhibits TNFRSF14 signaling and downregulates NK cell antitumor functions. High tumor expression of *TNFRSF14* and *CD160*, but not *TNFSF14*, *LTA*, or *BTLA*, was associated with improved BLCA patient prognosis, suggesting that the TNFRSF14–CD160 interaction, like KLRK1, is a prominent pathway of NK cell tumor surveillance in BLCA. In support of this, the expressions of *TNFRSF14*, *CD160*, and *KLRK1* were all strongly positively correlated with the IL2NK phenotype compared with other immune cell phenotypes in BLCA tumors.

Overall, these data strongly suggest that the NKG2D/NKG2D-L and TNFRSF14/CD160 pathways play a prominent role in the immune surveillance of BLCA tumors by IL-2-expanded NK cells. Our results show that high tumor abundance of the IL2NK phenotype and transcripts for the *KLRK1*, *CD160*, and *TNFRSF14* receptors are associated with improved survival, whereas high tumor abundance of the SPANK and *PDGFD* transcripts is associated with poor survival. Collectively, these data may be optimized to improve BLCA patient prognosis and risk stratification in the clinic. Interestingly, TNFRSF14 is also expressed by many tumor cells including BLCA, and TNFRSF14 ligation can induce BLCA cell apoptosis (48). Consequently, how the TNFRSF14–CD160 interaction might occur between NK cells and tumor cells in the BLCA tumor microenvironment to promote antitumor immunity and favorably impact patient prognosis remains unclear. Such information may help determine if the TNFRSF14–CD160 and NKG2D–NKG2D-L pathways can cooperate for NK cell cytotoxicity of BLCA tumor cells and whether these immune surveillance pathways can be targeted for in BLCA patients, e.g., using recombinant approaches to enhance NKG2D recognition of NKG2D-L expressing BLCA cells (106) or blocking negative regulators of TNFRSF14 activation, such as BTLA (47).

## Data Availability Statement

The original contributions presented in the study are included in the article/**Supplementary Material**. Further inquiries can be directed to the corresponding authors.

## Author Contributions

YS, MK, and SM performed the analyses. YP, SM, and AB designed and directed the research. YS, MK, AS, and AB wrote the manuscript. All authors contributed to the article and approved the submitted version.

## Funding

This work was funded by a MRFF research acceleration grant APP1162217 and a University of Melbourne Ph.D. scholarship awarded to YS. SM is funded by the Lorenzo and Pamela Galli Charitable Trust.

## Conflict of Interest

The authors declare that the research was conducted in the absence of any commercial or financial relationships that could be construed as a potential conflict of interest.

## Publisher’s Note

All claims expressed in this article are solely those of the authors and do not necessarily represent those of their affiliated organizations, or those of the publisher, the editors and the reviewers. Any product that may be evaluated in this article, or claim that may be made by its manufacturer, is not guaranteed or endorsed by the publisher.
