# Supplementary material for: A Transcriptional Signature of IL-2 Expanded Natural Killer Cells Predicts More Favorable Prognosis in Bladder Cancer
Source: Front Immunol. 2021 Nov 10;12:724107. doi: 10.3389/fimmu.2021.724107 (PMC8631443; doi:10.3389/fimmu.2021.724107)
Supplement: Supplementary file 2 [file Presentation_1.pptx]

## Slide 1
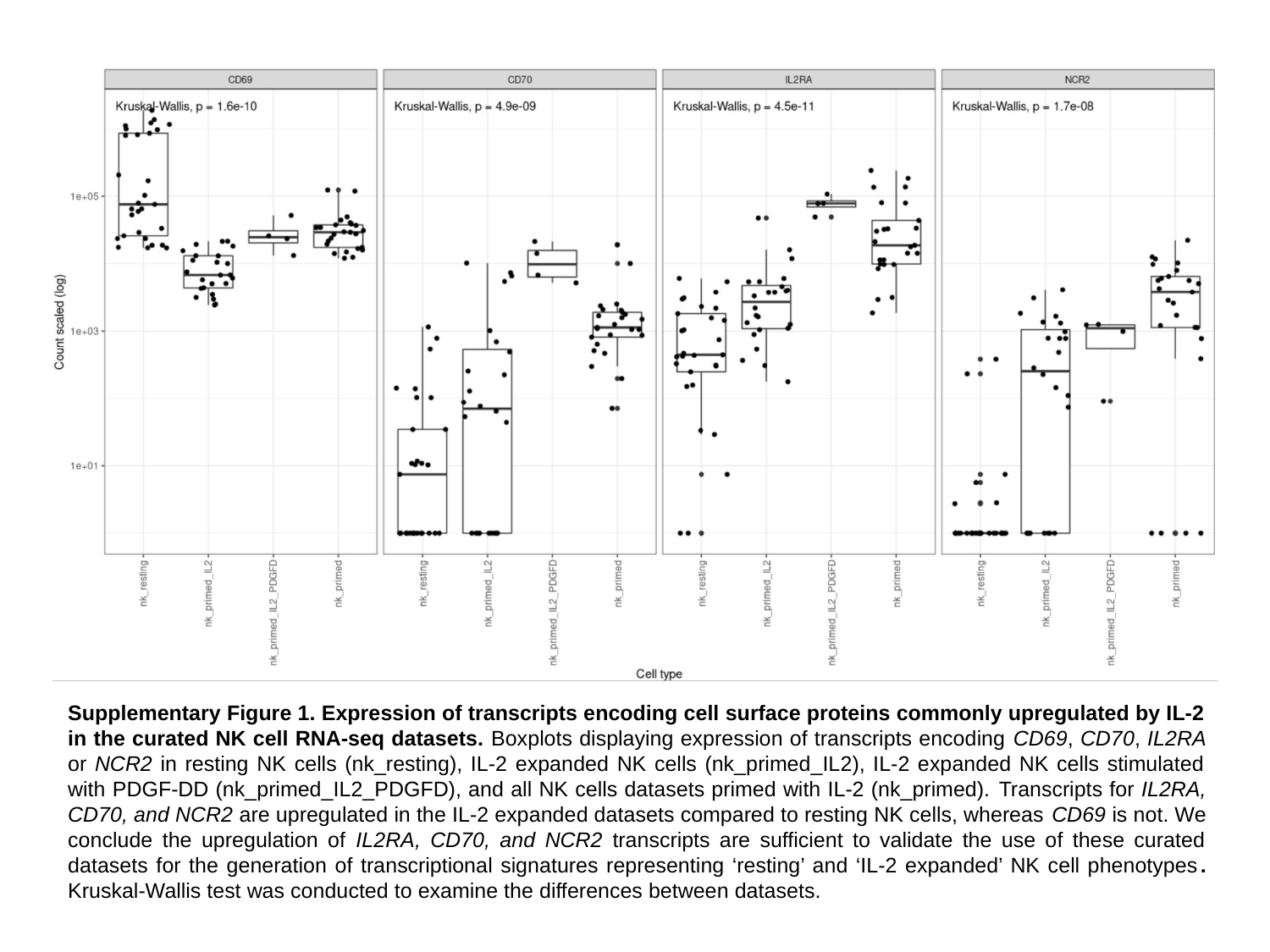

Supplementary Figure 1. Expression of transcripts encoding cell surface proteins commonly upregulated by IL-2 in the curated NK cell RNA-seq datasets. Boxplots displaying expression of transcripts encoding CD69, CD70, IL2RA or NCR2 in resting NK cells (nk_resting), IL-2 expanded NK cells (nk_primed_IL2), IL-2 expanded NK cells stimulated with PDGF-DD (nk_primed_IL2_PDGFD), and all NK cells datasets primed with IL-2 (nk_primed). Transcripts for IL2RA, CD70, and NCR2 are upregulated in the IL-2 expanded datasets compared to resting NK cells, whereas CD69 is not. We conclude the upregulation of IL2RA, CD70, and NCR2 transcripts are sufficient to validate the use of these curated datasets for the generation of transcriptional signatures representing ‘resting’ and ‘IL-2 expanded’ NK cell phenotypes. Kruskal-Wallis test was conducted to examine the differences between datasets.

## Slide 2
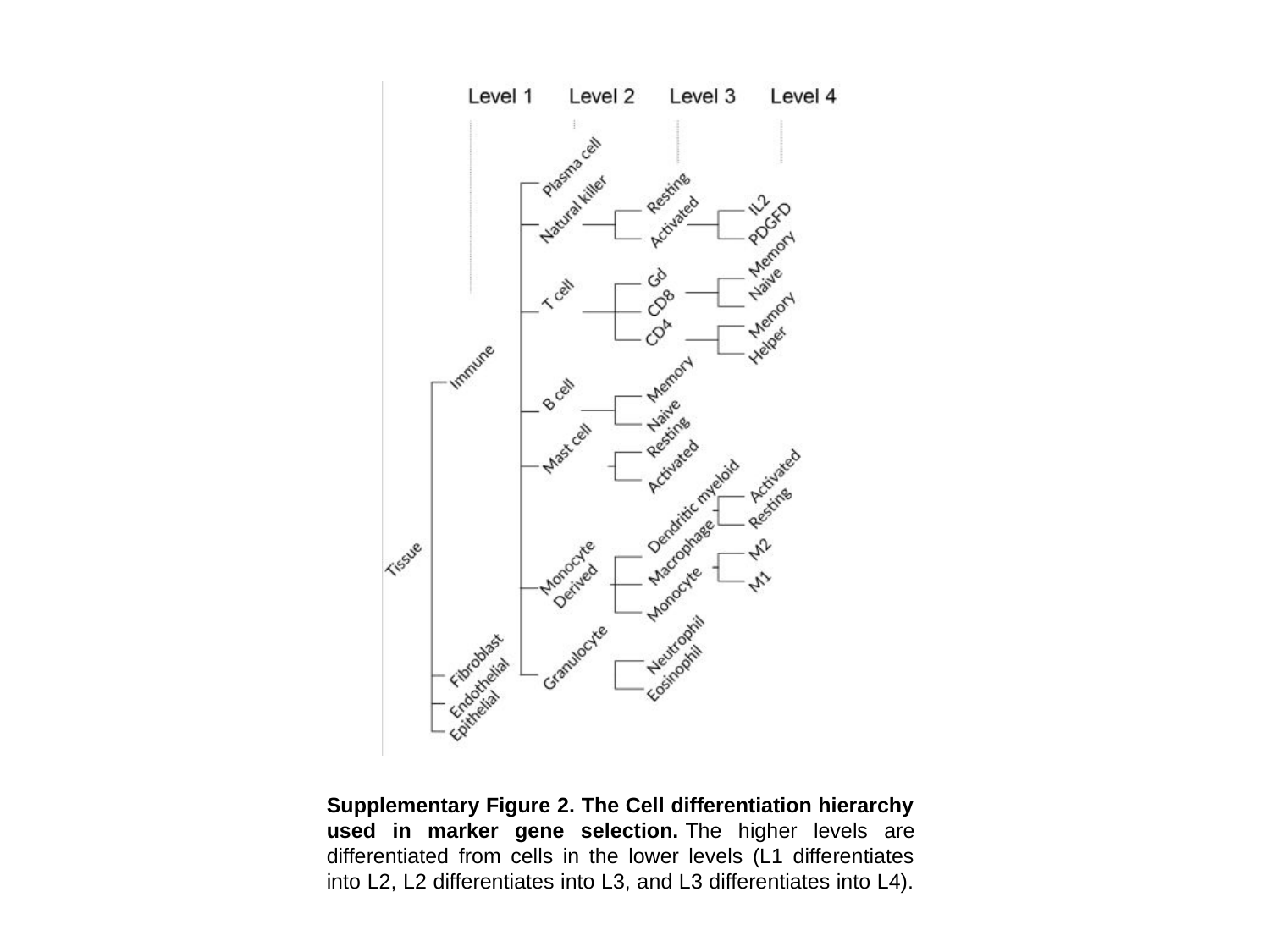

Supplementary Figure 2. The Cell differentiation hierarchy used in marker gene selection. The higher levels are differentiated from cells in the lower levels (L1 differentiates into L2, L2 differentiates into L3, and L3 differentiates into L4).

## Slide 3
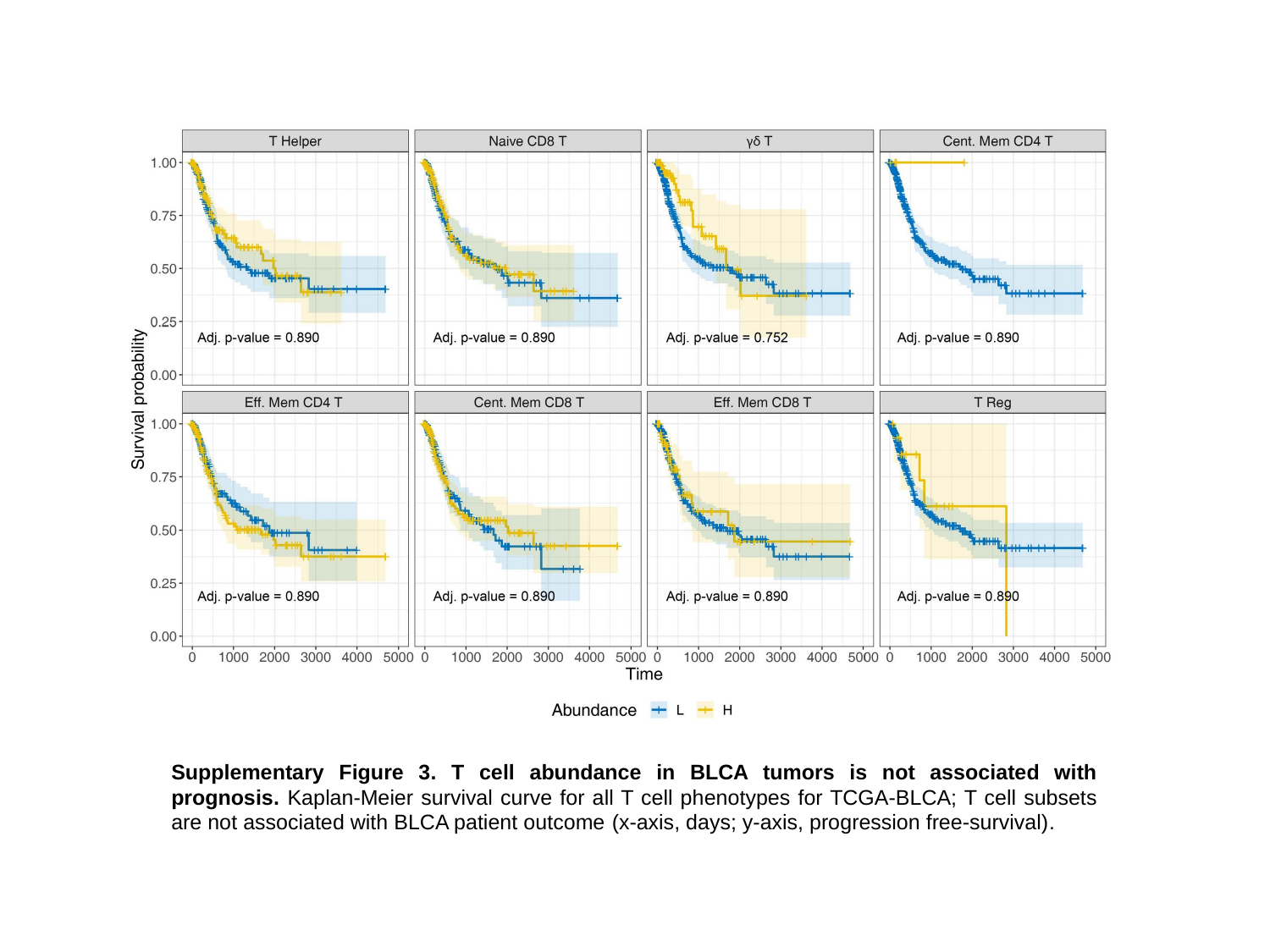

Supplementary Figure 3. T cell abundance in BLCA tumors is not associated with prognosis. Kaplan-Meier survival curve for all T cell phenotypes for TCGA-BLCA; T cell subsets are not associated with BLCA patient outcome (x-axis, days; y-axis, progression free-survival).

## Slide 4
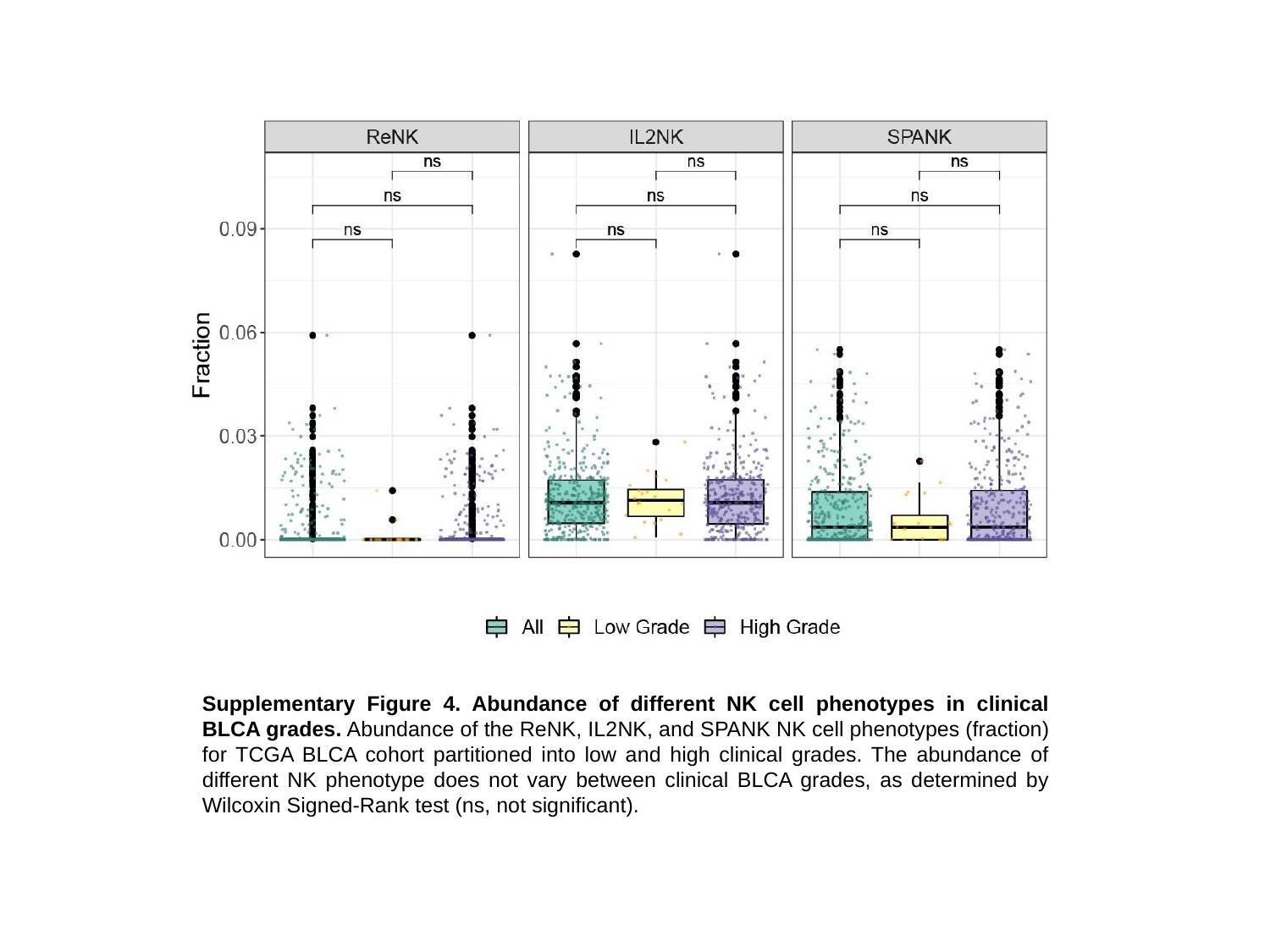

Supplementary Figure 4. Abundance of different NK cell phenotypes in clinical BLCA grades. Abundance of the ReNK, IL2NK, and SPANK NK cell phenotypes (fraction) for TCGA BLCA cohort partitioned into low and high clinical grades. The abundance of different NK phenotype does not vary between clinical BLCA grades, as determined by Wilcoxin Signed-Rank test (ns, not significant).

## Slide 5
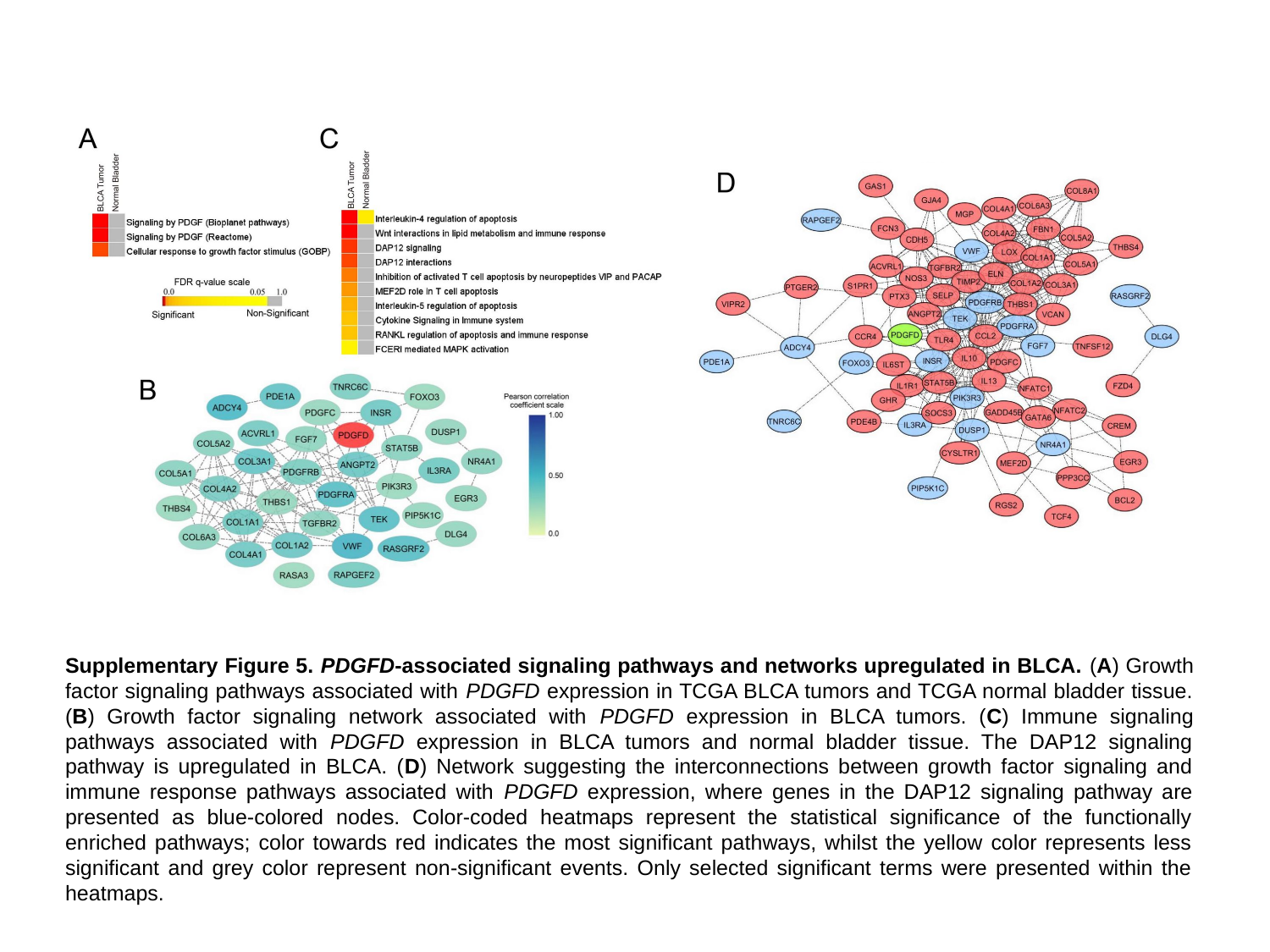

Supplementary Figure 5. PDGFD-associated signaling pathways and networks upregulated in BLCA. (A) Growth factor signaling pathways associated with PDGFD expression in TCGA BLCA tumors and TCGA normal bladder tissue. (B) Growth factor signaling network associated with PDGFD expression in BLCA tumors. (C) Immune signaling pathways associated with PDGFD expression in BLCA tumors and normal bladder tissue. The DAP12 signaling pathway is upregulated in BLCA. (D) Network suggesting the interconnections between growth factor signaling and immune response pathways associated with PDGFD expression, where genes in the DAP12 signaling pathway are presented as blue-colored nodes. Color-coded heatmaps represent the statistical significance of the functionally enriched pathways; color towards red indicates the most significant pathways, whilst the yellow color represents less significant and grey color represent non-significant events. Only selected significant terms were presented within the heatmaps.

## Slide 6
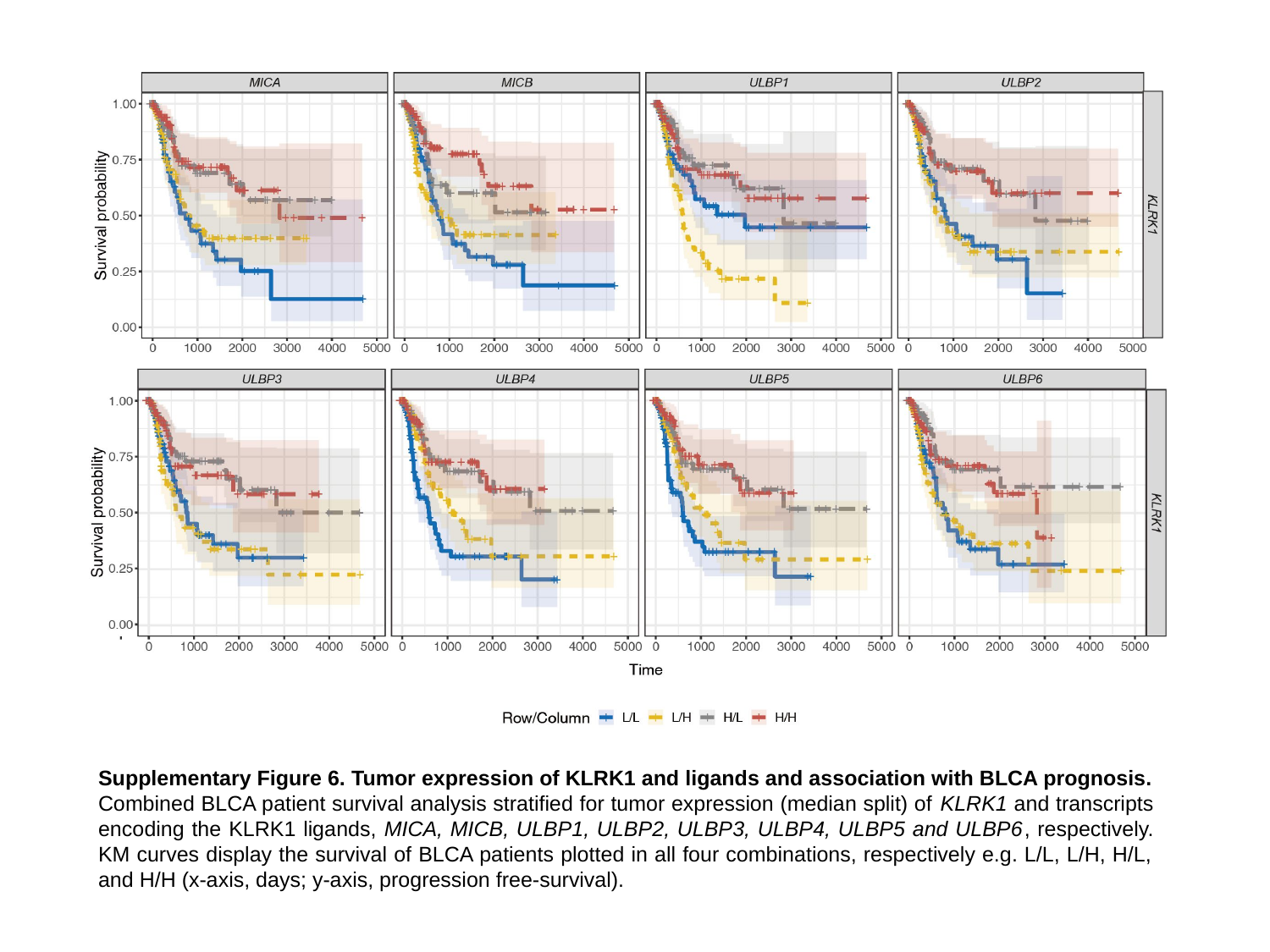

Supplementary Figure 6. Tumor expression of KLRK1 and ligands and association with BLCA prognosis. Combined BLCA patient survival analysis stratified for tumor expression (median split) of KLRK1 and transcripts encoding the KLRK1 ligands, MICA, MICB, ULBP1, ULBP2, ULBP3, ULBP4, ULBP5 and ULBP6, respectively. KM curves display the survival of BLCA patients plotted in all four combinations, respectively e.g. L/L, L/H, H/L, and H/H (x-axis, days; y-axis, progression free-survival).

## Slide 7
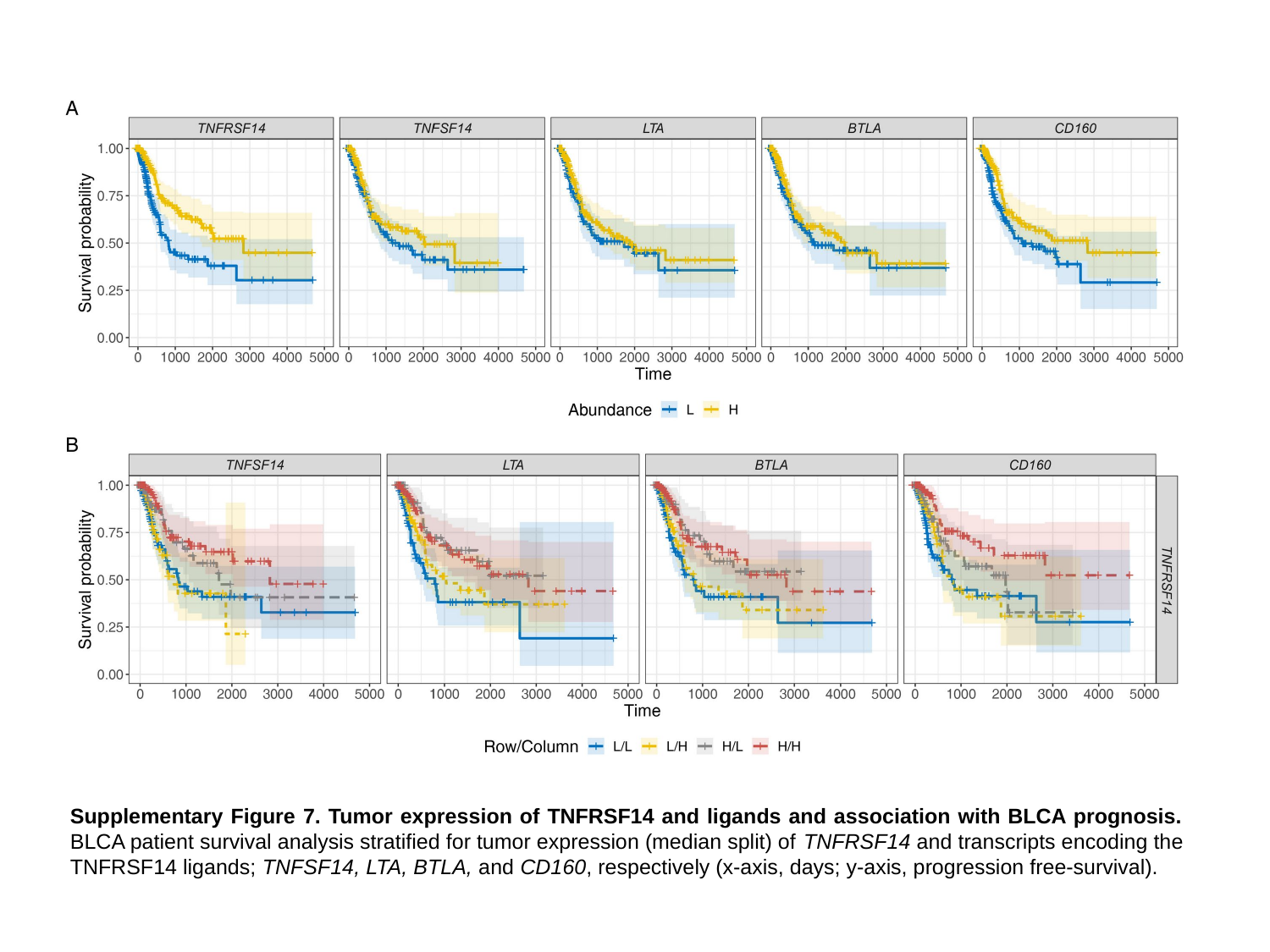

Supplementary Figure 7. Tumor expression of TNFRSF14 and ligands and association with BLCA prognosis. BLCA patient survival analysis stratified for tumor expression (median split) of TNFRSF14 and transcripts encoding the TNFRSF14 ligands; TNFSF14, LTA, BTLA, and CD160, respectively (x-axis, days; y-axis, progression free-survival).
