# Supplementary material for: A Transcriptional Signature of IL-2 Expanded Natural Killer Cells Predicts More Favorable Prognosis in Bladder Cancer
Source: Front Immunol. 2021 Nov 10;12:724107. doi: 10.3389/fimmu.2021.724107 (PMC8631443; doi:10.3389/fimmu.2021.724107)
Supplement: Supplementary file 3 [file Presentation_2.pptx]

## Slide 1
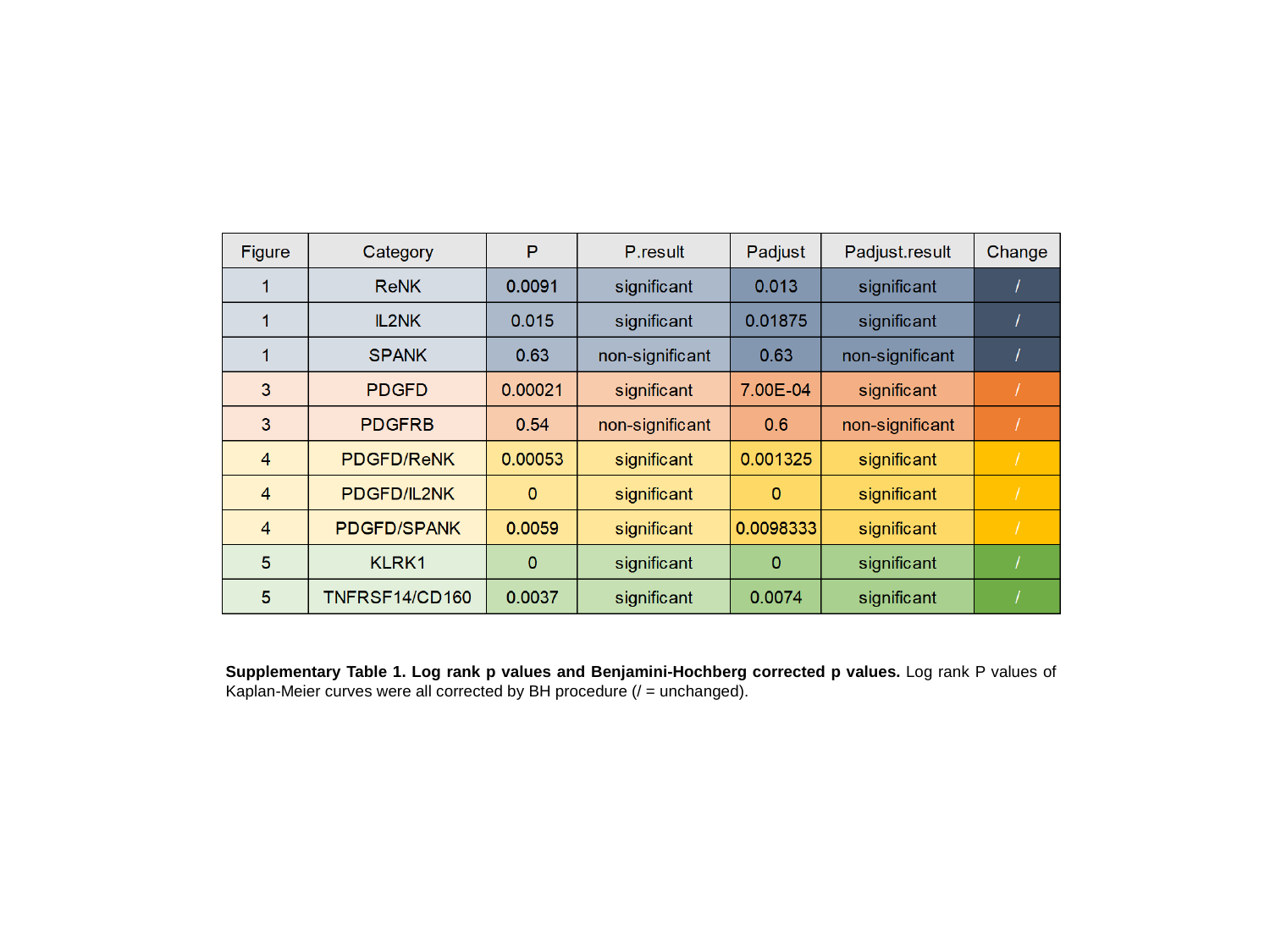

Supplementary Table 1. Log rank p values and Benjamini-Hochberg corrected p values. Log rank P values of Kaplan-Meier curves were all corrected by BH procedure (/ = unchanged).
